# Establishment of an Efficient and Flexible Genetic Manipulation Platform Based on a Fosmid Library for Rapid Generation of Recombinant Pseudorabies Virus

**DOI:** 10.3389/fmicb.2018.02132

**Published:** 2018-09-05

**Authors:** Mo Zhou, Muhammad Abid, Hang Yin, Hongxia Wu, Teshale Teklue, Hua-Ji Qiu, Yuan Sun

**Affiliations:** State Key Laboratory of Veterinary Biotechnology, Harbin Veterinary Research Institute, Chinese Academy of Agricultural Sciences, Harbin, China

**Keywords:** pseudorabies virus, fosmid library, full-length genome assemble, genetic manipulation platform, recombinant PRV

## Abstract

Conventional genetic engineering of pseudorabies virus (PRV) is essentially based on homologous recombination or bacterial artificial chromosome. However, these techniques require multiple plaque purification, which is labor-intensive and time-consuming. The aim of the present study was to develop an efficient, direct, and flexible genetic manipulation platform for PRV. To this end, the PRV genomic DNA was extracted from purified PRV virions and sheared into approximately 30–45-kb DNA fragments. After end-blunting and phosphorylation, the DNA fragments were separated by pulsed-field gel electrophoresis, the recovered DNA fragments were inserted into the cloning-ready fosmids. The fosmids were then transformed into *Escherichia coli* and selected clones were end-sequenced for full-length genome assembly. Overlapping fosmid combinations that cover the complete genome of PRV were directly transfected into Vero cells and PRV was rescued. The morphology and one-step growth curve of the rescued virus were indistinguishable from those of the parent virus. Based on this system, a recombinant PRV expressing enhanced green fluorescent protein fused with the VP26 gene was generated within 2 weeks, and this recombinant virus can be used to observe the capsid transport in axons. The new genetic manipulation platform developed in the present study is an efficient, flexible, and stable method for the study of the PRV life cycle and development of novel vaccines.

## Introduction

Pseudorabies (PR), also known as Aujeszky’s disease, is caused by pseudorabies virus (PRV) in the *Herpesviridae* family, which mainly affects swine and occasionally transmitted from pigs to cattle, sheep, goats, dogs, and cats (Masot et al., 2017; Zhou et al., 2017). Pigs are the only known natural reservoir for the virus. PRV has the ability to produce latent or clinically inapparent infections, which is transmitted between infected and non-infected pigs by nose-to-nose contact (Smith and Enquist, 2000; Pomeranz et al., 2017). The mortality in piglets <1 month of age approaches to 100%. A virulent PRV variant has emerged and become prevalent in China since 2011. The disease caused by this PRV variant is characterized by neurological signs and high mortality among newborn piglets (Wang et al., 2016, 2017).

PRV is a linear, double-stranded DNA virus of about 143-kb and consists of unique long (UL) region, unique short (US), internal repeat short (IRS), and terminal repeat short (TRS) (Pomeranz et al., 2017). The genome contains at least 70 open reading frames (ORFs) that encode 70–100 viral proteins, including structural proteins, virulence-related proteins and replicase (Pomeranz et al., 2005; An et al., 2013). The marked progress in molecular biotechnology has significantly contributed to the study of other viruses’ replication and vaccine. However, due to the large size of the PRV genome, the gene modification remains a difficult task. Earlier on, the recombinant PRV was generated by homologous recombination in permissive cells. Bacterial artificial chromosome (BAC) was used later and allowed cloning and manipulation of the whole genome in *Escherichia coli* (Lerma et al., 2016). The BAC system was more efficient than homologous recombination; however, from previous reports (Gu et al., 2015; Guo et al., 2016), it can be noticed that generation of recombinant BAC construct is time-consuming and labor-intensive due to several rounds of plaque purification and homologous recombination. However, generation of fosmid library is more efficient and minimizes the need of the above-mentioned steps.

The CopyControl cloning system of pCC1^TM^ Vector has a similar backbone to BAC and contains both a single-copy and the high-copy *ori*V origin of replication. Therefore, this system combines the clone stability afforded by single-copy cloning with the advantages of high DNA yields obtained by high-copy vector (Kim et al., 2003; Cunningham et al., 2006). Fosmids have been proved to have a high structural stability and found to maintain human DNA effectively even after 100 generations of bacterial growth, which was used for constructing stable libraries from complex genomes (Magrini et al., 2004; Zhang et al., 2007). A fosmid library is prepared by extracting the genomic DNA from the target organism, generating random genomic DNA fragments and cloning them into the fosmid vector (De Tomaso and Weissman, 2003; Moon and Magor, 2004). Therefore, construction of the infectious clones of large DNA viruses based on the fosmid library could alleviate the above drawbacks of BAC and allow manipulations of the viral genome more efficiently.

In this study, we constructed a fosmid library for the PRV-TJ strain, and an infectious progeny virus (rPRV-TJ) was rescued by directly transfecting fosmid sets into Vero cells. Moreover, a reporter virus (rPRV-VP26-EGFP) stably expressing enhanced green fluorescent protein (EGFP) was generated robustly via the Red/ET recombination. This study provides a foundation for rapid and accurate modification of the PRV genome. Meanwhile, the genetic manipulation of PRV based on the fosmid library also opens an exciting possibility and applicability for engineering other large DNA viruses in dissecting and probing genes of unknown functions.

## Materials and Methods

### Virus Strain and Cells

The PRV-TJ strain (GenBank accession number: KJ789182.1) was isolated from a pig farm with a PR outbreak in Tianjin, China in 2012 and stored at -70°C and propagated in the porcine kidney 15 (PK-15) cell line (Luo et al., 2014). PK-15 and Vero cells were obtained from China Center for Type Culture Collection (CCTCC, Wuhan, China) and maintained at 37°C with 5% CO_2_ in Dulbecco’s modified Eagle’s medium (DMEM) (Thermo-Fisher Scientific, Carlsbad, CA, United States) supplemented with 10% fetal bovine serum (Gibco, Grand Island, NY, United States). Dorsal root ganglions (DRGs) were isolated from newborn mice and cultured in Neurobasal medium (Gibco) supplemented with 100 ng/ml nerve growth factor 2.5S (Invitrogen), 2% B-27 (Gibco) and 1% penicillin and streptomycin with 2 mM glutamine (Invitrogen). The Animal Ethics Committee approval number is Heilongjiang-SYXK-2006-032. We conducted all the experiments in Biosafety Level II laboratory following strict biosecurity measures according to instructions of Harbin Veterinary Research Institute.

### Extraction of High-Quality PRV Genomic DNA and Construction of a Fosmid Library Covering the Full-Length Genome of PRV

The PRV-TJ strain propagated in PK-15 cell line was used to isolate genome for fosmid library construction according to the method described previously (Smith and Enquist, 1999). In brief, 10 75 cm^2^ flasks of confluent PK-15 cells were infected with the PRV-TJ strain at a multiplicity of infection (MOI) of 5. The cells were then incubated at 37°C for 15 h, and harvested by scraping. The scrapped cells were then washed twice with phosphate-buffered saline (PBS). The final cell pellet was re-suspended in 10 ml of LCM buffer (130 mM KCl, 30 mM Tris [pH 7.4], 5 mM MgCl_2_, 0.5 mM EDTA, 0.5% nonidet P-40 [NP-40], and 0.043% 2-mercaptoethanol). The virus particle was extracted with Freon from re-suspended pellet and then the nucleocapsid pellets were extracted by centrifugation through two LCM buffer-based glycerol step gradients (8 ml of 5% glycerol and 16 ml of 45% glycerol) at 26,000 rpm for 2.5 h at 4°C. The nucleocapsid pellets were used to extract the genome. DNA quality was assessed by NanoDrop^TM^ 2000 (Thermo Scientific) and transfection. For transfection, monolayer of Vero cells grown on 6-well-plate was washed with PBS and then 2 ml of DMEM without antibiotics was added in each well for 1 h. The Vero cells were transfected with 2 μg of PRV genomic DNA using X-treme GENE HP DNA transfection reagent (Roche). The ratio of X-treme GENE HP DNA transfection reagent (μl) to PRV genomic DNA (μg) was 1:1.

Twenty microgram of PRV genomic DNA (at a concentration of 500 ng/μl) was pipetted 800 times with a 200-μl tip to shear the genomic DNA into approximately 30–45-kb fragments. To determine proper shearing, 1 μl of sheared DNA was analyzed on a 1% gold agarose gel by pulsed-field gel electrophoresis (PFGE) using Fosmid Control DNA (Epicentre) and a Lambda DNA-Mono Cut Mix (New England BioLabs) as size marker. In order to generate 5′-phosphorylated DNA, 20 μg of sheared genomic DNA was end repaired using the End-Repairing Enzyme Mix according to the CopyControl^TM^ Fosmid Library Production Kit. Following end repair, the genomic DNA was size selected on a low-melting point agarose gel by PFGE. DNA fragments ranging from 33 to 48-kb in size were excised from the gel and recovered using GELase (Epicentre) according to the instruction manual. The recovered fragments were then ligated into the pCC1FOS cloning-ready vector at room temperature for 4 h. The ligation mixture was subsequently packaged using MaxPlax Lambda Packaging Extracts. Ten microliter of the packaged phage was then added to 100 μl of EPI300-T1 cells. The infected EPI300-T1 cells were spread on the LB ager plate containing 12.5 μg/ml chloramphenicol.

### Fosmid Sequencing and Full-Length Genome Assembly

The resulting number of clones for a complete fosmid library covering the entire PRV genome is 92. Therefore, 200 clones were randomly picked and cultured overnight in 5-ml LB liquid medium containing 12.5 μg/ml chloramphenicol and 50 μl of auto-induction solution (Epicentre). The fosmids were extracted using ZR BAC DNA Miniprep Kit (Zymo Research). Fosmid end-sequencing was performed using pCC1FOS sequencing primers. The inserted sequences of all the fosmids were screened by BLAST. All sequences with 100% identity were screened out from the data set. The fosmids that cover the complete PRV genome were used to assemble the full-length genome.

### Rescue of the Recombinant PRV

Ten overlapping fosmid combinations (each group containing five fosmids) that cover the complete PRV genome were used for virus rescue. Briefly, 80–90% confluent Vero cells grown on 10-cm plates were washed with PBS and then cultured with 10 ml of DMEM medium without antibiotics for 1 h. Meanwhile, five overlapping fosmids in each group (2 μg each) were gently mixed with 30 μl of X-treme GENE HP DNA transfection reagent in 1 ml of DMEM and incubated at room temperature for 20 min. The mixture was added into the above-mentioned Vero cell monolayers and the transfected cells were incubated at 37°C with 5% CO_2_. At 3 days post-transfection (dpt), the cell supernatant was harvested when most cells showed cytopathic effects (CPEs) for virus passaging and further characterization. Vero cells transfected with the fosmid sets missing one fosmid served as negative control.

### Immunofluorescence Assay (IFA)

To confirm the rescued virus (rPRV-TJ), a swine anti-PRV serum derived from the PRV-TJ strain-infected pigs was used as primary antibody in indirect immunofluorescence analysis. PK-15 cells were seeded in 96-well plates and cultured in DMEM containing 5% FBS. The confluent cell monolayers were infected with serially 10-fold diluted rPRV-TJ for 36 h. The cells were fixed with ethanol for 30 min at -30°C, followed by incubation with swine anti-PRV sera (diluted 1:300 with PBS) for 2 h at 37°C and then with Alexa 488-conjugated goat anti-pig IgG (Thermo Fisher Scientific) (1:1,000) for 1 h at 37°C. Images were captured using an Olympus CK40 microscope.

### PCR

To confirm the integrity of rPRV-TJ, the gB and gE genes were detected by PCR using the genome of rPRV-TJ as a template, the genome of PRV-TJ was used as the positive control. The specific primers for gE (5′-TGGCTCTGCGTGCTGTGCTC-3′ and 5′-CATTCGTCACTTCCGGTTTC-3′) and gB (5′-GGGGTTGGACAGGAAGGACACCA-3′ and 5′-AACCAGCTGCACGCTCAA-3′) were used. TaKaRa LA Taq^TM^ with GC Buffer (TaKaRa) was used for PCR amplification. The reaction mixtures were performed in a final volume of 20 μl, containing 2 μl dNTP mixture, 1.0 μM concentration of each primer, 10 μl 2× GC Buffer I, 2 μl of virus DNA sample, and 0.25 μl of LA Taq. Reactions were conducted in an automated DNA thermal cycler (Bio-Rad, United States). The thermo-cycling condition was denaturation for 5 min at 95°C, followed by 35 cycles that each consisted of a denaturation step at 95°C for 30 s, an annealing step at 60°C for 30 s, and an extension at 72°C for 1 min, and the final extension at 72°C for 10 min.

### Pulsed-Field Gel Electrophoresis

The genomic DNA (10 μg) of the rescued or parental virus was digested with *Kpn*I, *Nco*I, and *Pst*I, respectively, for 5 h at 37°C. The reaction was transferred to 70°C for 10 min to inactivate the restriction enzymes. The digested samples were analyzed by PFGE in a 1% (w/v) gold agarose gel in 0.5× TBE buffer at 6 V/cm and 14°C for 16 h. The λ DNA-Mono Cut Mix was used as standard.

### Electron Microscopy

Vero cells were infected with rPRV-TJ and PRV-TJ and harvested at 48 h post-infection (hpi). Cell culture medium was centrifuged at 3,000 × *g* for 10 min, the supernatant was collected and centrifuged at 10,000 × *g* for 10 min, and then the pellet was resuspended in PBS. The sample was negatively stained with 2% phosphotungstic acid, the morphology of the rescued virus was observed under electron microscope and PRV-TJ particles as positive control to compare the morphology.

### Plaque Assay

rPRV-TJ and PRV-TJ were serially 10-fold diluted in DMEM. One hundred microliter of diluted sample was inoculated onto Vero cell monolayers in 12-well culture plates. After incubation for 1 h at 37°C, the monolayers were washed twice with DMEM and overlaid with 2 ml of DMEM containing 1% low melting point agarose. The plaque-forming units (PFUs) were determined at 5 days post-infection.

### Replication Kinetics of the Rescued PRV

The virus titers of rPRV-TJ and PRV-TJ were determined according to the Reed-Muench method. PK-15 cells cultured in a 24-well plate were infected with rPRV-TJ and PRV-TJ at an MOI of 10 and incubated on ice for 1 h. Thereafter, the inoculum was replaced with pre-warmed fresh medium and cells were further incubated for 1 h at 37°C and rinsed for 2 min with citrate buffer (pH 3.0) to inactivate any unabsorbed virus. Then fresh medium was added and the cells were incubated at 37°C in 5% CO_2_. The cultures were harvested at 0, 4, 8, 12, 16, 20, 24, 28, 32, 36, 48, 60, and 72 hpi. The titers of all the collected samples were determined in duplicates on monolayers of PK-15 cells, and the average of each was calculated as described previously (Luo et al., 2014).

### Generation of a Recombinant PRV Using the Fosmid Library and Red/ET System

Capsid assembly occurs in the nucleus of infected cells, initially with a spherical pro-capsid precursor built around a protein scaffold that matures into a DNA-containing capsid. VP26 is one of the first herpesvirus proteins to be fused with a fluorescent protein. Capsid-tagged virus mutants have been used to study capsid transport, intra-nuclear capsid dynamics, and nuclear egress (Desai and Person, 1998; Hogue et al., 2015). Therefore, in this study, the EGFP gene was inserted between the second and third codons of the VP26 gene by Counter Selection BAC Modification Kit (Gene Bridges, Berkeley, CA, United States) according to the manufacturer’s instructions. In the first step, the Red/ET expression plasmid (pRed/ET) and the fosmid were co-transformed into competent *E. coli* DH10B cells by electroporation. In the next step, the antibiotic selectable cassette (*rpsL-neo*) flanked by the homology arms was generated by PCR amplification with specific primers in **Table 1** and inserted into the target site of the fosmid by the Red/ET-mediated recombination. To fuse the EGFP gene with the VP26 gene, the electro-component cells were prepared from the cells containing modified fosmid carrying a *rpsL-neo* cassette. In advance, the linear DNA fragment of the EGFP gene with homology arms was amplified with specific primers in **Table 1**. The EGFP gene flanked by two oligonucleotide homology arms was transformed into the prepared electro-component cells to replace the *rpsL-neo* cassette by the Red/ET-mediated recombination. The modified VP26 ORFs were amplified and sequenced. Finally, the modified fosmid plus the other fosmids were transfected into Vero cells to rescue the virus. Vero cells transfected with the fosmid set missing one fosmid served as a negative control and those transfected with the unmodified fosmid set as a positive control, and the recombinant virus expressing the VP26-EGFP fusion protein was rescued.

**Table 1:** Primers for Red/ET recombination.

Names	Sequences (5′–3′)	Target genes	Insert position
F-VP26-rpsL	GCGCGCGGGGGCGCGCACAGACGCGCGCTCCCCGCCGAGCCATCATGTCC*GGCCTGGTGATGATGGCGGGATCG*	rpsL	After the second codon of VP26
R-VP26-rpsL	GCGCCCTCGAGCGTCTGCGCGGTGATCGTCCGGGGATTGTTCGGGTCGAA*TCAGAAGAACTCGTCAAGAAGGCG*		
F-VP26-EGFP	GCGCGCGGGGGCGCGCACAGACGCGCGCTCCCCGCCGAGCCATCATGTCC*GTGAGCAAGGGCGAGGAGC*	EGFP	After the second codon of VP26
R-VP26-EGFP	GCGCCCTCGAGCGTCTGCGCGGTGATCGTCCGGGGATTGTTCGGGTCGAA*TCTAGATCCGGTGGATCCCG*		
F-identify rpsL	CATCATCCTGAACATGCG		
R-identify rpsL	GCTGCTGTAGTCGCTGGTG		


### Characterization of the Recombinant Virus

To evaluate the genetic stability of the reporter virus containing the EGFP gene, rPRV-VP26-EGFP was passaged in PK-15 cells for 20 generations. The essential genes (gB and gE) were amplified using the genome of the rescued virus as a template according to the above-mentioned method, and the inserted gene (EGFP) was also amplified with the specific primers (5′-CATCATCCTGAACATGCG-3′ and 5′-CATCATCCTGAACATGCG-3′). Replication kinetics and PFU of the rescued PRV were analyzed according to the above mentioned method.

### Infection of Neurons With rPRV-VP26-EGFP

Microfluidic device is a useful tool for neuroscience research, which can separate the neuron and axons (Taylor et al., 2003; Harris et al., 2007). In this study, the neuron microfluidic device was used to observe the capsid transportation between neurons and axons. The microfluidic device can separate the soma and axonal side of DRGs. The DRGs were isolated from 2-day neonatal BALB/C mice and loaded into the axonal soma of the devices as described in previous report (Harris et al., 2007). One day after seeding, 5 mM arabinofuranoside (AraC; Sigma-Aldrich) was added for at least 2 days to eliminate non-neuronal cells. Neurons were cultured for around 5 days, the axons grown and flown through to the axon side. The rPRV-VP26-EGFP was infected to the soma side at an MOI of 5, and thus, the axon side could not contact rPRV-VP26-EGFP. Therefore, we excluded the possibility that the virus entered both neurons and axons. The green fluorescent capsids were observed at 12 hpi under a fluorescence microscope.

## Results

### Extraction of High-Quality PRV Genomic DNA

The concentration of PRV genomic DNA was determined by Thermo Scientific NanoDrop^TM^ 2000. The concentration was 692.5 ng/μl and A260/A280 was 1.80. The full-length genome of PRV was used to transfect Vero cells using X-treme GENE HP DNA transfection reagent. At 24 h post-transfection, CPEs were observed in most transfected cells. The cell culture supernatant was harvested and used to inoculate PK-15 cells and the CPE became obvious (data not shown). Therefore, the concentration and quality of genomic DNA could thus satisfy the needs of fosmid library construction.

### Generation of the Fosmid Library for PRV

**FIGURE 1:**
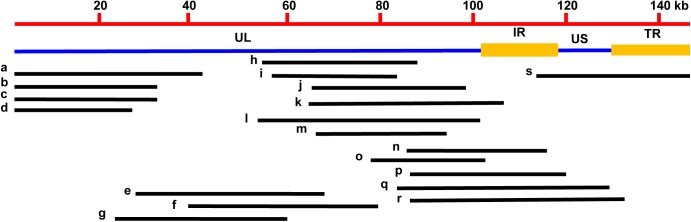
Fosmid library construction for the PRV-TJ strain. Genomic structure of PRV and the five fosmid DNAs used for rescue of PRV. Numbers show the location of each fosmid fragment in the PRV genome.

A total of 200 clones were randomly picked from the fosmid library for end-sequencing, representing more than twofold coverage of PRV genome. A total of 180 clones contained DNA fragments of PRV-TJ, a majority of which contains inserts of 30–40-kb. A fosmid library covering the complete genome of PRV was established. Nineteen fosmids were selected for generating the fosmid-combinations that cover the entire genome of PRV (**Table 2**). Ten sets of overlapping fosmid-combinations were prepared to rescue the recombinant PRV, each consisting of five overlapping fosmids (**Figure 1** and **Table 3**).

**Table 2:** Fosmids that cover the entire genome of PRV.

Fosmid	Location in genome (nt)	Size (bp)	Fosmid	Location in genome (nt)	Size (bp)
a	1-41633	41,633	k	66341-111030	4,4690
b	1-35363	35,363	l	49114-89118	4,0005
c	1-32129	32,129	m	64783-95747	3,0947
d	1-29834	37,955	n	87260-116855	2,9596
e	29544-67498	32,055	o	62988-100570	3,7583
f	37942-69996	35,778	p	82459-124194	4,1736
g	23721-59498	31,544	q	81316-117252	3,5937
h	55457-87000	34,390	r	86680-125105	3,8426
i	48838-83227	34,390	s	113582-143642	3,00061
j	66874-98904	32,031			


**Table 3:** Fosmid combinations that cover the entire genome of PRV.

Group	Combinations	CPE	Group	Combinations	CPE
1	b + e + h+p+s	+	6	b + e + i+q+s	+
2	a + f + k+r+s	-	7	a + f + j+q+s	-
3	c + g + l+r+s	+	8	a + f + m+n+s	+
4	a + f + o+q+s	+	9	d + g + h+p+s	-
5	b + e + h+q+s	+	10	d + g + l+q+s	-


### Rescue of PRV From Overlapping Fosmids and Characterization of the Rescued PRV

**FIGURE 2:**
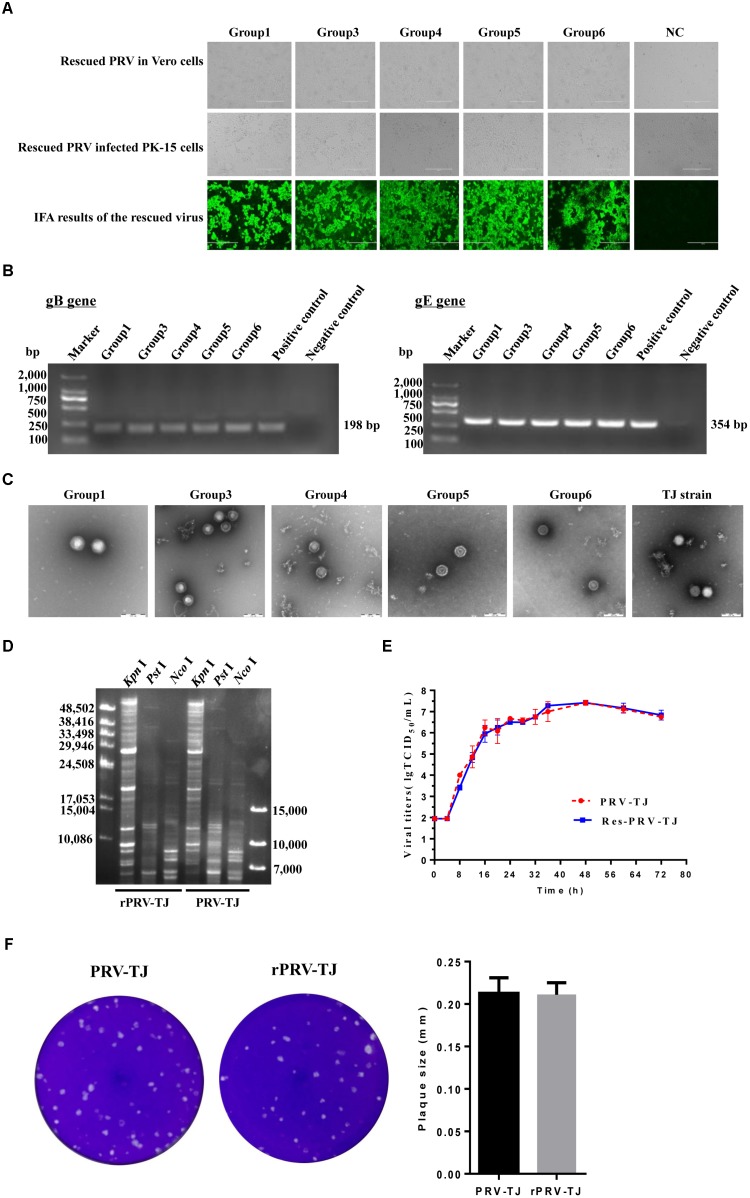
Identification of the rescued PRV. **(A)** Transfection of the Vero cells with the fosmid combinations, the cytopathic effects (CPEs) was observed. Vero cells transfected with the fosmid set missing one fosmid was used as negative control. The culture supernatants of transfected cells was collected and used to infect PK-15 cells, the CPEs were also observed. The rescued PRV was detected by IFA with an anti-PRV serum. **(B)** PCR amplification of the gB and gE genes. The gB and gE genes were amplified using the genome of the rescued virus as a template. The genome of the parental PRV was used as a positive control. The irrelevant genome was used as a negative control. **(C)** Transmission electron of viral DNA of the rescued and the parent PRV. PRV-TJ particles were used as positive control. Scale bars are presented. **(D)** The restriction profiles of the rescued and the parent PRV in 1% agarose. The genome of the rescued and the parent PRV was digested with *Kpn*I, *Nco*I, and *Pst*I. **(E)** One-step growth curves of the rescued and the parent PRV. PK-15 cells cultured in 24-well plates were infected with the rescued or the parent PRV at a multiplicity of infection (MOI) of 10, after incubated on ice and rinsed with citrate buffer, the virus was harvested from both the medium and cells at 0, 4, 8, 12, 16, 20, 24, 28, 32, 36, 48, 60, and 72 h post-infection (hpi), and titers were determined. Titration was performed in triplicates; error bars represent standard errors of the mean. **(F)** The plaque size of the rescued and the parent PRV. The rescued and the parent PRV were 10-fold serial diluted at a titer at which a single plaque could be formed. The experiments were performed in triplicates, and the representative results were shown.

Ten sets of fosmid were transfected into Vero cells to rescue the virus. The concentration of each fosmid was determined in ng/μl and then volume was adjusted according to 2 μg for each fosmid. Each group contain five overlapping fosmids, 2 μg of each fosmid was gently mixed with 30 μl of X-treme GENE HP DNA transfection reagent for transfection. CPEs were observed in Vero cells at 2 dpt from sets 1, 3, 4, 5, and 6, but not from other sets and the negative control. The PK-15 cells infected with rPRV-TJ were assayed by IFA using swine anti-PRV sera at 24 hpi (**Figure 2A**). The expected bands of the gB and gE genes were amplified from the genomic DNA of rPRV-TJ (**Figure 2B**). Under electron microscope, the rPRV-TJ particles showed similar morphology to that of the parental virus with an apparently external envelope (**Figure 2C**). The genome of rPRV-TJ was digested with *Kpn*I, *Nco*I and *Pst*I, and analyzed by PFGE. The digestion patterns of *Kpn*I and *Nco*I are 100%, according to our observations, the *Pst*I digestion pattern is also similar and the difference may be due to band intensity (**Figure 2D**). The replication kinetics and plaque morphology of rPRV-TJ and the parental virus had no significant difference (**Figures 2E,F**).

### Generation of rPRV-TJ Expressing EGFP

**FIGURE 3:**
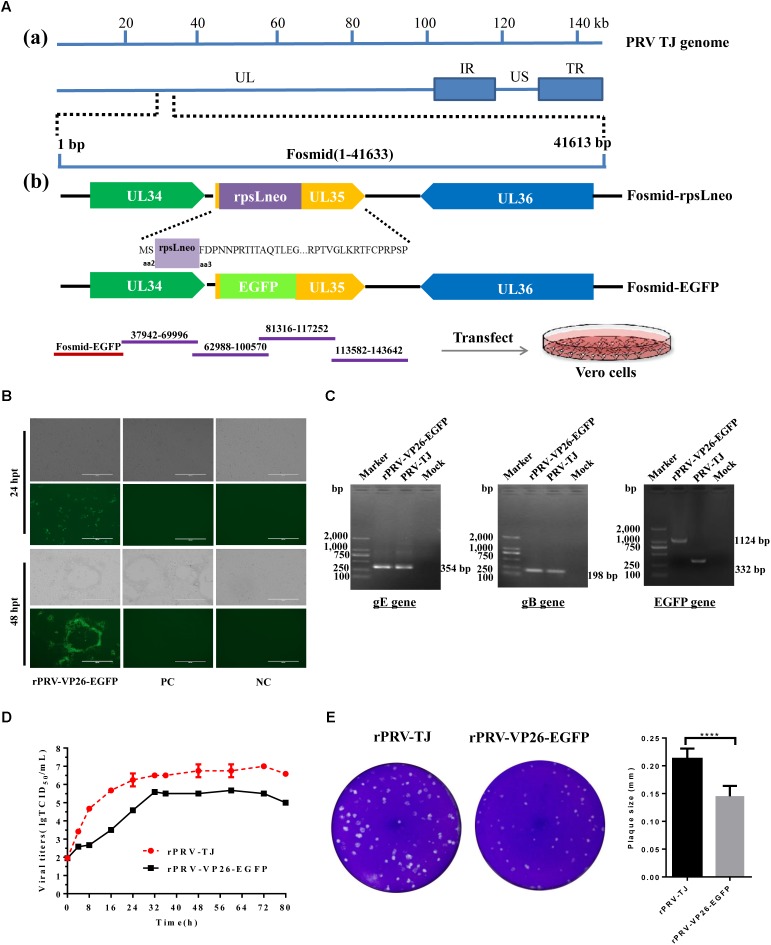
Generation of rPRV-VP26-EGFP. **(A)** Schematic representation of Fosmid(1-41,633) modification for the generation of a recombinant PRV-TJ strain expressing the EGFP gene. *a*: The flow diagram of the PRV genome and the position of Fosmid(1-41,633). *b*: Construction procedure of the intermediate fosmids and Fosmid-EGFP. For fosmid modification, the antibiotics-selectable cassette (*rpsL-neo*) flanked by two oligonucleotide homology arms was inserted between the second and third amino acids of VP26 by the Red/ET-mediated recombination. The EGFP gene flanked by the same homology arms was used to replace the *rpsL-neo* cassette to generate Fosmid-EGFP. For whole genome assembly, Fosmid-EGFP and other fosmids in Group 4 were transfected into Vero cells to generate a recombinant PRV expressing the EGFP gene. **(B)** Fosmid-EGFP and other fosmids in Group 4 were transfected into Vero cells to rescue the virus. Vero cells that were transfected with the fosmid set missing one fosmid was served as negative control and Vero cells transfected with the un-modified fosmid set as positive control. The images were taken at 24 and 72 hpi. **(C)** PCR amplification of the gB, gE, and EGFP genes. The gB, gE, and EGFP genes were amplified using the genome of the rescued virus as a template. The genome of the parental virus was used as a positive control. The externally located primers, which across the insertion site, were used to amplify the EGFP gene. **(D)** The replication kinetics of rPRV-TJ and rPRV-VP26-EGFP. The virus titers were calculated at 0, 4, 8, 12, 16, 20, 24, 28, 32, 36, 48, 60, and 72 hpi. **(E)** Plaque size of rPRV-TJ and rPRV-VP26-EGFP. The virus was 10-fold diluted at a titer at which single plaques could be formed. The experiments were performed in triplicates, and representative results were shown.

VP26 is the small capsid protein expressed early during replication, tagging VP26 is helpful to visualize alpha herpesvirus capsids under fluorescence microscopy (Hogue et al., 2015). Therefore, the VP26 gene was used for tagging the capsid protein by fusion with the coding sequence of EGFP in this study. To rescue rPRV-VP26-EGFP, we modified Fosmid(1-41,633) that harbors the VP26 gene (**Figure 3A**). The resulting modified fosmid and other fosmids in the combination were transfected into Vero cells. After 2 days CPEs and green fluorescence were detected (**Figure 3B**). CPEs were observed in the Vero cells transfected with the complete fosmid set but not with the set with one fosmid missing. Expected bands of the gB, gE, and EGFP genes were amplified from the genomic DNA of the rescued rPRV-VP26-EGFP (**Figure 3C**). The genomic DNA of PRV-TJ was used as the positive control. The externally located primers that cross the insertion site were used to amplify the EGFP gene. The growth kinetics of rPRV-VP26-EGFP was delayed compared with rPRV-TJ, the rPRV-VP26-EGFP has around 1 log defect in the peak virus titer relative to rPRV-TJ (**Figure 3D**). The plaque size of the rescued virus in Vero cells was also smaller than that of rPRV-TJ (**Figure 3E**).

### Visualization of the EGFP-Tagged Capsids in Neuron Bodies and Axons

**FIGURE 4:**
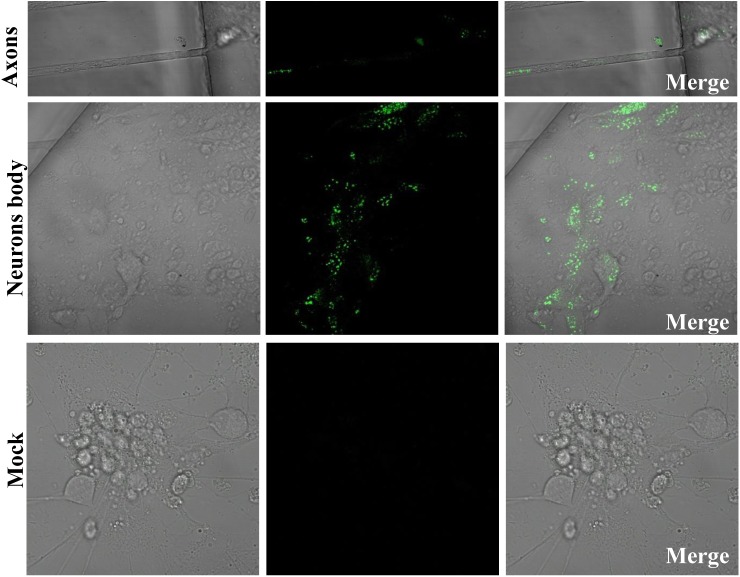
rPRV-VP26-EGFP infection in neuron cells. Dorsal root ganglions (DRGs) were plated in the main channel of Microfluidics. After 5 days, DRGs were infected with rPRV-VP26-EGFP at an MOI of 5. Fluorescent images were taken 12 hpi, the green fluorescent capsids accumulated in cell bodies and transport between neuron bodies and axons.

We generated a reporter PRV virus containing EGFP to monitor virus moving both toward the cell body (retrograde) and away from the cell body (anterograde), and thus can facilitate to study virus transport, intra-nuclear capsid dynamics and nuclear egress. Therefore, using the EGFP fused PRV-TJ VP26 we were able to analyze the virus transport in axons. The soma side was infected with the rPRV-VP26-EGFP, and the EGFP signals were imaged at 12 hpi, the EGFP-tagged capsid transported from the soma side to the axons that in the connecting region of soma and axonal side, there was no EGFP signal in the mock cells (**Figure 4**). The results indicate that rPRV-VP26-EGFP allows visualization of the EGFP-tagged capsid transport between neuron bodies and axons.

## Discussion

Generating recombinant PRV using traditional methods, such as plasmid transfection plus virus co-infection, often are inefficient, labor-intensive, and time-consuming due to the need of cloning and purification processes. In addition, the insertion of a selection marker is another tedious and time-consuming process. Infectious BACs of herpesviruses are powerful tools for genetic manipulation (Tobler and Fraefel, 2015; Close et al., 2017). However, construction of BAC clones usually takes several months and the presence of BAC vector sequence in the viral genomes often causes genetic and phenotypic alterations (Zhao et al., 2008; Zhou et al., 2010). Alternatively, fosmid library provides a powerful platform for rescue of viruses in recent studies (Liu et al., 2011, 2016; Li et al., 2016). This system allows unbiased inclusion of only viral genomic DNA fragments and seamless cloning without any genetic scar in comparison to fragments generated by restriction enzymes. Therefore, in order to improve genetic manipulation platform for PRV, in this study, a PRV fosmid library was constructed. High-quality PRV genomic DNA preparation is a critical step to construct a fosmid library. Preparation of high molecular weight DNA (around 40-kb) is also an important step as well as the basis for constructing a high quality library. Therefore, the quality of PRV genomic DNA was assessed by transfection to make sure the integrity of the PRV genomic DNA for the fosmid library, after that the genomic DNA was sheared into approximately 30–45-kb fragments, separated by PFGE, excised from gel and recovered. Thus, the average insert size for the fosmid library is 30–40-kb, and the number of clones with an insert is >90% and a high quality fosmid library was generated in this study.

Different sets of fosmids that cover the complete genome of PRV were used to rescue recombinant PRVs. Ten overlapping fosmid combinations were used to rescue virus. However, some combinations were successful to rescue virus, whereas others did not produce CPEs at 3 dpt. The overlapping region of each combination was different, which may be the reason of the variable efficiency among these combinations to rescue the virus. The aim of this study was to screen the fosmid combinations that could produce CPEs in short time period, only sets 1, 3, 4, 5, and 6 could rescue the virus within 3 dpt, other combinations were also monitored up to 5 days and no CPEs were observed, so we did not proceed to further steps.

The fosmid library-based genetic manipulation platform for PRV offers several advantages over the conventional technology. First, PRV genome was randomly fragmented into suitable 40-kb DNA fragments for cloning into the pCC1FOS vector is much simpler and far less time-consuming because there is no need to isolate high molecular weight DNA, or perform partial restriction enzyme digestions. Furthermore, high cloning efficiency of fosmid also makes it easy to achieve full genome coverage. Therefore, fosmid library facilitates the generation of infectious PRV. Second, fosmid vector maintains the clones as single copy, thereby enhancing insert stability. Meanwhile, the fosmid vector contains an inducible, high-copy origin oriV, which increases copy number for higher yields in the presence of an inducer without compromising insert stability. Third, the established methodology is flexible to rescue recombinant viruses from overlapping fragments of cloned viral DNA, which is based on minimal sequence modification in bacteria and allows the modification of any essential genes of PRV by Red/ET recombination. The modification of individual 40-kb-fosmid was more convenient than the oversized BAC DNA constructs. Fourth, the construction of homology arms and plaque purification are not required in this system. Therefore, the use of the fosmid library greatly reduces the time and labor for generating recombinant PRVs.

In this study, we constructed a fosmid library of PRV and rescued the virus by transfecting overlapping fosmids into Vero cells. The typical virus biological characteristics such as morphology and one-step growth curve analyses revealed that the rescued virus was indistinguishable from the parental virus. The recombinant virus expressing EGFP fused to VP26 was generated based on the fosmid library-based genetic manipulation platform, which allows further monitoring the pathogenicity of PRV *in vivo* and *in vitro.* Furthermore, rPRV-VP26-EGFP allows us to monitor visually the localization of the virus at various stages of infection. We found that rPRV-VP26-EGFP caused about 10-fold defect in single-step virus replication (**Figure 3D**). Some studies also reported the effect of fluorescent protein fusions to VP26 on virus replication kinetics, cell-to-cell spread and pathogenesis *in vivo* (Krautwald et al., 2008; Hogue et al., 2015). The possible explanation to these differences might be the insertion of EGFP gene affect capsid assembly. In addition, the plaque sizes of the recombinants were smaller than those of PRV-TJ (**Figure 3E**). Some reports indicated that fluorescent protein fuses to VP26 affects cell-to-cell spread of the recombinant virus. The smaller plaque size may indicate the cell-to-cell spread ability of the recombinant virus become lower. Therefore, the smaller plaque size may correlate with lower replication capability of rPRV-VP26-EGFP.

In summary, this genetic manipulation platform provides an opportunity to explore the biology of PRV in depth. Similarly, the method of fosmid constructing platform can be extended to other large double-stranded DNA viruses. This platform will be directly used for the development of novel bivalent, trivalent and marker vaccines. We believe that any newly emerged PRV strain and other DNA viruses can be manipulated using this platform, and possibly vaccines could be developed in a short time period.

## Author Contributions

MZ, YS, and H-JQ designed the study. MZ wrote the manuscript. MZ, MA, HY, HW, and TT performed the experiments. All authors reviewed the manuscript.

## Conflict of Interest Statement

The authors declare that the research was conducted in the absence of any commercial or financial relationships that could be construed as a potential conflict of interest.

## References

[B1] An T. Q. Peng J. M. Tian Z. J. Zhao H. Y. Li N. Liu Y. M. (2013). Pseudorabies virus variant in bartha-K61-vaccinated pigs, China. *Emerg. Infect. Dis.* 19 1749–1755. 10.3201/eid1911.130177 24188614 PMC3837674

[B2] Close W. L. Bhandari A. Hojeij M. Pellett P. E. (2017). Generation of a novel human cytomegalovirus bacterial artificial chromosome tailored for transduction of exogenous sequences. *Virus Res.* 242 66–78. 10.1016/j.virusres.2017.09.007 28912069

[B3] Cunningham C. Hikima J. Jenny M. J. Chapman R. W. Fang G. C. Saski C. (2006). New resources for marine genomics: bacterial artificial chromosome libraries for the eastern and Pacific oysters (*Crassostrea virginica* and *C. gigas).* *Mar. Biotechnol.* 8 521–533. 10.1007/s10126-006-6013-9 16896533

[B4] Desai P. Person S. (1998). Incorporation of the green fluorescent protein into the herpes simplex virus type 1 capsid. *J. Virol.* 72 7563–7568. 9696854 10.1128/jvi.72.9.7563-7568.1998 PMC110002

[B5] De Tomaso A. W. Weissman I. L. (2003). Construction and characterization of large-insert genomic libraries (BAC and fosmid) from the ascidian *Botryllus schlosseri* and initial physical mapping of a histocompatibility locus. *Mar. Biotechnol.* 5 103–115. 10.1007/s10126-002-0071-1 12876644

[B6] Gu Z. Dong J. Wang J. Hou C. Sun H. Yang W. (2015). A novel inactivated gE/gI deleted pseudorabies virus (PRV) vaccine completely protects pigs from an emerged variant PRV challenge. *Virus Res.* 195 57–63. 10.1016/j.virusres.2014.09.003 25240533

[B7] Guo J. C. Tang Y. D. Zhao K. Wang T. Y. Liu J. T. Gao J. C. (2016). Highly efficient CRISPR/Cas9-mediated homologous recombination promotes the rapid generation of bacterial artificial chromosomes of pseudorabies virus. *Front. Microbiol.* 7:2110. 10.3389/fmicb.2016.02110 28066407 PMC5179515

[B8] Harris J. Lee H. Vahidi B. Tu C. Cribbs D. Cotman C. (2007). Non-plasma bonding of PDMS for inexpensive fabrication of microfluidic devices. *J. Vis. Exp.* 9:410. 10.3791/410 18989450 PMC2566327

[B9] Hogue I. B. Bosse J. B. Engel E. A. Scherer J. Hu J. R. Del Rio T. Enquist L. W. (2015). Fluorescent protein approaches in alpha herpesvirus research. *Viruses* 7 5933–5961. 10.3390/v7112915 26610544 PMC4664988

[B10] Kim C. G. Fujiyama A. Saitou N. (2003). Construction of a gorilla fosmid library and its PCR screening system. *Genomics* 82 571–575. 10.1016/S0888-7543(03)00174-5 14559214

[B11] Krautwald M. Maresch C. Klupp B. G. Fuchs W. Mettenleiter T. C. (2008). Deletion or green fluorescent protein tagging of the pUL35 capsid component of pseudorabies virus impairs virus replication in cell culture and neuroinvasion in mice. *J. Gen. Virol.* 89 1346–1351. 10.1099/vir.0.83652-0 18474549

[B12] Lerma L. Muñoz A. L. Wagner S. Dinu M. Martín B. Tabarés E. (2016). Construction of recombinant pseudorabies viruses by using PRV BACs deficient in IE180 or pac sequences: application of vBAC90D recombinant virus to production of PRV amplicons. *Virus Res.* 213 274–282. 10.1016/j.virusres.2015.11.028 26756577

[B13] Li K. Liu Y. Liu C. Gao L. Zhang Y. Cui H. (2016). Recombinant marek’s disease virus type 1 provides full protection against very virulent marek’s and infectious bursal disease viruses in chickens. *Sci. Rep.* 6:39263. 10.1038/srep39263 27982090 PMC5159867

[B14] Liu J. Chen P. Jiang Y. Wu L. Zeng X. Tian G. (2011). A duck enteritis virus-vectored bivalent live vaccine provides fast and complete protection against H5N1 avian influenza virus infection in ducks. *J. Virol.* 85 10989–10998. 10.1128/JVI.05420-11 21865383 PMC3194986

[B15] Liu Y. Li K. Gao Y. Gao L. Zhong L. Zhang Y. (2016). Recombinant marek’s disease virus as a vector-based vaccine against Avian leukosis virus subgroup J in chicken. *Viruses* 8:301. 10.3390/v8110301 27827933 PMC5127015

[B16] Luo Y. Li N. Cong X. Wang C. H. Du M. Li L. (2014). Pathogenicity and genomic characterization of a pseudorabies virus variant isolated from bartha-K61-vaccinated swine population in China. *Vet. Microbiol.* 174 107–115. 10.1016/j.vetmic.2014.09.003 25293398

[B17] Magrini V. Warren W. C. Wallis J. Goldman W. E. Xu J. Mardis E. R. (2004). Fosmid-based physical mapping of the *Histoplasma capsulatum* genome. *Genome Res.* 14 1603–1609. 10.1101/gr.2361404 15289478 PMC509269

[B18] Masot A. J. Gil M. Risco D. Jiménez O. M. Núñez J. I. Redondo E. (2017). Pseudorabies virus infection (Aujeszky’s disease) in an Iberian lynx (*Lynx pardinus*) in Spain: a case report. *BMC Vet. Res.* 13:6. 10.1186/s12917-016-0938-7 28056966 PMC5217549

[B19] Moon D. A. Magor K. E. (2004). Construction and characterization of a fosmid library for comparative analysis of the duck genome. *Anim. Genet.* 35 417–418. 10.1111/j.1365-2052.2004.01177.x 15373752

[B20] Pomeranz L. E. Ekstrand M. I. Latcha K. N. Smith G. A. Enquist L. W. Friedman J. M. (2017). Gene expression profiling with cre-conditional pseudorabies virus reveals a subset of midbrain neurons that participate in reward circuitry. *J. Neurosci.* 37 4128–4144. 10.1523/JNEUROSCI.3193-16.2017 28283558 PMC5391685

[B21] Pomeranz L. E. Reynolds A. E. Hengartner C. J. (2005). Molecular biology of pseudorabies virus: impact on neurovirology and veterinary medicine. *Microbiol. Mol. Biol. Rev.* 69 462–500. 10.1128/MMBR.69.3.462-500.2005 16148307 PMC1197806

[B22] Smith G. A. Enquist L. W. (1999). Construction and transposon mutagenesis in *Escherichia coli* of a full-length infectious clone of pseudorabies virus, an alphaherpesvirus. *J. Virol.* 73 6405–6414. 10400733 10.1128/jvi.73.8.6405-6414.1999 PMC112720

[B23] Smith G. A. Enquist L. W. (2000). A self-recombining bacterial artificial chromosome and its application for analysis of herpesvirus pathogenesis. *Proc. Natl. Acad. Sci. U.S.A.* 97 4873–4878. 10.1073/pnas.080502497 10781094 PMC18325

[B24] Taylor A. M. Rhee S. W. Tu C. H. Cribbs D. H. Cotman C. W. Jeon N. L. (2003). Microfluidic multicompartment device for neuroscience research. *Langmuir* 19 1551–1556. 10.1021/la026417v 20725530 PMC2923462

[B25] Tobler K. Fraefel C. (2015). Infectious delivery of alphaherpesvirus bacterial artificial chromosomes. *Methods Mol. Biol.* 1227 217–230. 10.1007/978-1-4939-1652-8_10 25239748

[B26] Wang J. Guo R. Qiao Y. Xu M. Wang Z. Liu Y. (2016). An inactivated gE-deleted pseudorabies vaccine provides complete clinical protection and reduces virus shedding against challenge by a chinese pseudorabies variant. *BMC Vet. Res.* 12:277. 10.1186/s12917-016-0897-z 27923365 PMC5142131

[B27] Wang X. Wu C. X. Song X. R. Chen H. C. Liu Z. F. (2017). Comparison of pseudorabies virus China reference strain with emerging variants reveals independent virus evolution within specific geographic regions. *Virology* 506 92–98. 10.1016/j.virol.2017.03.013 28363130

[B28] Zhang L. Bao Z. Cheng J. Li H. Huang X. Wang S. (2007). Fosmid library construction and initial analysis of end sequences in Zhikong scallop (*Chlamys farreri*). *Mar. Biotechnol.* 9 606–612. 10.1007/s10126-007-9014-4 17605073

[B29] Zhao Y. Petherbridge L. Smith L. P. Baigent S. Nair V. (2008). Self-excision of the BAC sequences from the recombinant marek’s disease virus genome increases replication and pathogenicity. *Virol. J.* 5:19. 10.1186/1743-422X-5-19 18230192 PMC2248170

[B30] Zhou F. Li Q. Wong S. W. Gao S. J. (2010). Autoexcision of bacterial artificial chromosome facilitated by terminal repeat-mediated homologous recombination: a novel approach for generating traceless genetic mutants of herpesviruses. *J. Virol.* 84 2871–2880. 10.1128/JVI.01734-09 20071577 PMC2826046

[B31] Zhou J. Li S. Wang X. Zou M. Gao S. (2017). Bartha-k61 vaccine protects growing pigs against challenge with an emerging variant pseudorabies virus. *Vaccine* 35 1161–1166. 10.1016/j.vaccine.2017.01.003 28131396

